# Thyroidea Ima Artery in Cadavers of a Medical College in Nepal: A Descriptive Cross-sectional Study

**DOI:** 10.31729/jnma.8540

**Published:** 2024-04-30

**Authors:** Mina Jha, Shaligram Dhungel, Ashwini Kumar Jha, Sudeep Kumar Yadav

**Affiliations:** 1 Department of Anatomy, Janaki Medical College and Teaching Hospital, Janakpur Dham, Dhanusha, Nepal; 2 Department of Anatomy, Nepal Medical College and Teaching Hospital, Attarkhel, Kathmandu, Nepal; 3 Department of Surgery, Janaki Medical College and Teaching Hospital, Janakpur Dham, Dhanusha, Nepal

**Keywords:** *brachiocephalic trunk*, *cadavers*, *thyroid gland*

## Abstract

**Introduction:**

Thyroidea ima artery is a variant arterial branch of arch of aorta supplying the thyroid gland. Understanding the anatomic variances and correctly identifying the thyroidea ima artery is crucial to preventing serious complications both before and after neck surgery. The aim of this study was to find out the prevalence of thyroidea ima artery in cadavers of a medical college in Nepal.

**Methods:**

A descriptive cross-sectional study was carried out at the department of anatomy in Janaki Medical College, Dhanusha, Nepal from 27 December 2022 to 30 June 2023 after ethical clearence from the same institution. Origin of thyroidea ima artery was observed, recorded and photographed. Convenience sampling method was used. Data was analyzed using Microsoft Excel.

**Results:**

Out of 35 cadavers, thyroidea ima artery was present in 2 (5.71%), arising from brachiocephalic trunk just proximal to its bifurcation and there was absence of inferior thyroid artery.

**Conclusions:**

Findings from our study showed that thyroidea ima artery originated from brachiocephalic trunk with absence of inferior thyroid artery.

## INTRODUCTION

The thyroid gland is a highly vascularized endocrine gland, with an isthmus connecting the right and left lobe. The gland receives its arterial supply from a paired Superior and Inferior Thyroid arteries and occasionally from a variant artery, thyroidea ima artery.^1^ It can be encountered ascending from anterior of trachea, traversing the superior mediastinum and neck to reach the thyroid gland.^2^

According to anatomic variation reference books, it may affect 4-10% of individuals.^3^ However, only 0.4% of population was identified by Adachi; while other authors reported ranging from 1.5% to 12.2% of subject.^4,5^ As thyroidea ima artery is uncommon and have unpredictable origins, courses, and morphologies, they might complicate neck procedures.^6^

Knowledge of its course and variation could facilitate surgeons for blood less surgeries and avoid chances of complications during the procedures of neck. Since there aren't plenty of studies on the topic in the Nepalese population, thus study aimed to determine the prevalence of thyroidea ima artery in cadavers.

## METHODS

A descriptive cross-sectional study was conducted in the Anatomy Department of Janaki Medical College and Teaching Hospital, Ramdaiya, Dhanusha. The study was conducted from 27 December 2022 to 30 June 2023 after obtaining the ethical approval from the Institutional Review Committee of the same institution (Reference number: IRC/25/2079-80). In the study, cadavers (both male and female) used for routine dissection for the undergraduate medical course and the preserved specimens were included. Those with any surgical procedures performed in the thorax and neck and with any pathological conditions in neck were excluded from the study.

Dissection was performed to explore the mediastinum and neck. The fatty tissue and the pericardium covering the ascending aorta, arch of aorta with its branches and the great vessels were dissected out. Special attention was paid toward preserving vascular findings around the aortic arch and identifying any anomalies. Arterial branching of the brachiocephalic trunk, left common carotid artery, and left subclavian artery was observed for its anatomical relations and traced. Upon identification of a TIA or other vascular variation, other surrounding tissue were removed from the dissection plane. Observation was recorded in preformed proforma and photograph was taken.

Obtained data was entered and analyzed using Microsoft Excel.

## RESULTS

A total of 35 cadavers and specimens available at the anatomy department of Janaki Medical College and Teaching Hospital were dissected and examined for the presence of thyroidea ima artery (TIA). The thyroidea ima artery was present in two (5.72%) of cadavers. Among them 33 (94.28%) of the cadavers had normal branching pattern. The normal branching pattern showed brachiocephalic trunk, left common carotid artery and left subclavian artery from right to left. There was presence of superior thyroid artery in all the cadavers but there was absence of inferior thyroid artery in two cadavers where thyroidea ima artery was present. Among 35 cadavers, 20 (57.14%) were male and 15 were (42.86%) female (Table 1).

**Table 1:** Gender distribution of cadavers (n= 35).

Gender	n (%)
Male	20 (57.14)
Female	15 (42.86)

The presence of thyroidea ima artery was seen in one (2.86%) male and one (2.86%) female cadaver (Table 2).

**Table 2:** Origin of Thyroidea Ima Artery (n= 35).

Gender	Normal n (%)	Presence of TIA n (%)
Male	19 (54.28%)	1 (2.86%)
Female	14 (40%)	1 (2.86%)

In both cadavers, it was originating from the brachiocephalic trunk just proximal to its bifurcation into the right common carotid and subclavian artery. The brachiocephalic trunk arises from the arch of aorta. The thyroidea ima artery was supplying the inferior part and isthmus of the thyroid gland.

## DISCUSSION

In the present study, out of 35 cadavers, the presence of thyroidea ima artery was seen in 2 (5.72%) cadavers. The site of origin of the thyroidea ima artery in both cadavers was from the brachiocephalic trunk proximal to its bifurcation into the right common carotid and right subclavian artery showing the absence of right inferior thyroid artery. There was presence of right superior thyroid artery in all of cadavers. Similar finding reported in a study of embalmed cadavers in West Bengal population, showing 3.33% cases with Thyroidea Ima Artery arising from Arch of Aorta and Brachiocephalic Trunk.^7^

A study conducted on unclaimed bodies in Forensic Medicine department of a Medical College of Dhaka revealed the presence of Thyroidea ima artery in 10.52%, most commonly it was originated from brachiocephalic trunk, few from arch of aorta and right common carotid artery, which is higher than the present study but had similar site of origination from brachiocephalic trunk.^8^

A study conducted on 94 cadavers at Touro College of Osteopathic Medicine, New York found thyroidea ima artery in only one (1.06%) cadaver which is less than the present study.^2^

The thyroidea ima artery was first mentioned in literatures in 1772, also known as arteria thyroidea ima, accessory thyroid artery, Thyroidea ima artery of Neubauer, and lowest thyroid artery.^9^

Frequently observed and depicted as an immediate arterial offshoot of the brachiocephalic trunk, the arteria thyroidea ima may have its origin in smaller vessels like the pericardiophrenic artery and the thyrocervical trunk, or in other major arteries like the aortic arch, common carotid arteries, and subclavian arteries.^10,11^ Thyroidea ima artery can originate from internal thoracic artery, aortic arch, right common carotid artery, or brachiocephalic artery.^12^

The embryological genesis of the arteria thyroidea ima has been proposed to be associated with variable vascular differentiation during the fetal period and the descent of the thyroid, with varying mediastinal origins, lengths, and termination sites.^13,14^

Having a typical diameter of 3 to 5 millimeters, the thyroide a ima artery is classified as a tiny arterial artery. Its sources of origin are from bi g, high-pressure arteries and if hemostasis is not established quickly, there may be significant bleeding and blood loss.^15^ In cases where the patient has been stabilized otherwise, immediate transfer for hemostasis and arterial reconstruction has been demonstrated to enhance results.^2^

Because this study was conducted on small samples from a single centre, its findings might not apply to the whole population. To allow the real results to be generalized, comparable studies should be conducted with a larger sample size.

## CONCLUSIONS

Thyroidea ima artery injury can result during endovascular operations, tracheostomy procedures, and neck surgeries, and can cause bleeding during and after the treatment because of its highly variable origin and course. Furthermore, in the context of neck angiography, it can be interpreted incorrectly. As a result, experts in the field of neck surgery, endocrinology, otolaryngology, diagnostic radiology, endovascular intervention, vascular surgery, and anatomy must possess comprehensive understanding of this artery.
